# HIV takes double hit before entry

**DOI:** 10.1186/1741-7007-10-99

**Published:** 2012-12-07

**Authors:** Rogier W Sanders

**Affiliations:** 1 Laboratory of Experimental Virology, Department of Medical Microbiology, Center for Infection and Immunity Amsterdam (CINIMA), Academic Medical Center, University of Amsterdam, 1105 AZ Amsterdam, The Netherlands; 2 Department of Microbiology and Immunology, Weill Medical College of Cornell University, New York, NY 10065, USA

## Abstract

In the absence of a vaccine or a cure, identification of novel HIV-1 inhibitors remains important. A paper in *Retrovirology *describes a rationally designed bi-specific protein that irreversibly damages the viral envelope glycoprotein complex via a two-punch mechanism. In contrast to traditional drugs that inhibit essential steps in the viral life cycle at the cell surface or in the infected cells, this inhibitor cripples free virus in the absence of cells.

See research article: http://www.retrovirology.com/content/9/1/104

## Commentary

Antiviral drugs are potent at suppressing viral replication in HIV infected individuals. Unfortunately, current drug regimens cannot cure infected persons because of the establishment very early in infection of latent viral reservoirs that cannot be eliminated by conventional drugs. The continuous virus evolution and the danger of viral escape from the available drugs necessitate the search for novel inhibitors. HIV drugs can target a variety of processes of the viral life cycle that include viral entry into host cells, viral reverse transcription, integration into the host genome, and virus maturation.

**Figure 1:**
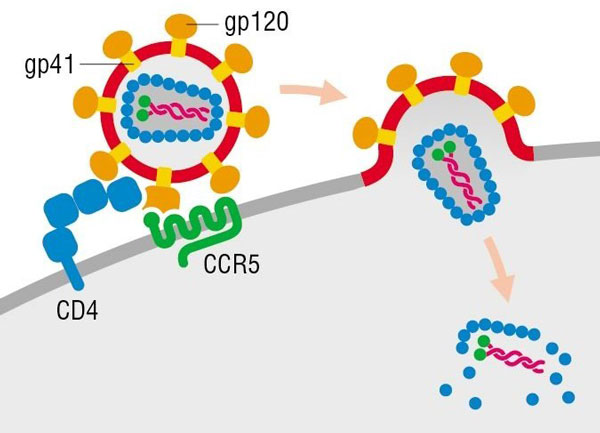
**HIV entry into a host cell**. Highly schematic diagram of viral entry. The trimeric gp120 subunit of the viral envelope glycoprotein complex (Env) binds CD4 on the target cell surface, triggering a conformational change that promotes interactions with chemokine receptors and activates the trimeric transmembrane subunit gp41 to mediate membrane fusion, so that the viral contents can enter the cell. Reproduced from DeFranco AL, Locksley RM, Robertson M: Immunity: The Immune Response in Infectious and Inflammatory Disease. London: New Science Press; 2007, with permission of Oxford University Press.

The HIV envelope glycoprotein complex (Env) is an intricate molecular machine that mediates the viral attachment to target cells and the fusion of viral with cellular membranes (Figure 1), and does so in a highly coordinated manner. Env has been compared with a mousetrap [1]: it is spring-loaded and snaps shut when touching an infectable target cell. This process occurs roughly in three phases, each of which can be inhibited by a class of entry inhibitors. The first event is the binding of the surface subunit gp120 to the primary receptor on the target cell, CD4, and this step can be inhibited by CD4 mimetics, though these have not proved effective enough for clinical use [2]. CD4 binding creates and exposes the binding site on gp120 for the second receptor, one of the chemokine receptors CCR5 or CXCR4. The binding to the co-receptor can be inhibited by co-receptor antagonists [3], an example of which is the CCR5-antagonist maraviroc, which has been in clinical use since 2007. CD4 binding also induces conformational changes in the transmembrane subunit gp41 that result in exposure of the hydrophobic fusion peptides. Insertion of the fusion peptides into the target membrane, followed by further conformational changes induced by binding to the co-receptor, culminates in membrane fusion and release of the viral genetic material into the cytoplasm. The CD4-induced, activated state of gp41 can be targeted by fusion inhibitors [4] and one such fusion inhibitor, enfuvirtide (T20), has been used to treat HIV-1 infected individuals since 2003. Owing to its poor bioavailability (it requires intravenous injection twice daily), and relatively expensive manufacturing (it is a peptide), the use of enfuvirtide is declining as it is superceded by cheaper alternative drugs that are orally bioavailable. Nevertheless, enfuvirtide saved many lives when it came on the market at a time when no alternative new drugs were available. Second and third generation enfuvirtide-like fusion inhibitors have been designed that are more potent, have better pharmacokinetic properties, and are less prone to viral escape [5]. One such fusion inhibitor is the peptide T1144.

Now a team led by Shibo Jiang has designed a novel bi-specific inhibitor, 2DLT, which essentially is a fusion protein of a soluble version of CD4 and the third generation fusion inhibitor T1144 [6]. As such it can inhibit the interaction of gp120 with CD4 as well as the conformational changes in gp41 that result in membrane fusion, resulting in HIV-1 inhibition at low nanomolar concentrations. While bi-specific and multispecific proteins are widely studied for use in cancer therapy, only a few bi-specific molecules have been designed for HIV-1. One such molecule, termed sCD4-17b, shares a CD4 mimetic component with 2DLT, but further contains an antibody fragment directed to an epitope on gp120 that is induced by CD4 binding and that overlaps with the co-receptor binding site [7]. A second approach uses bi-specific antibody molecules targeting two different epitopes, one on gp120 and one on gp41 [8]. Both sCD4-17 and the bi-specific antibodies result in low nanomolar inhibition of HIV similar to 2DLT.

The beauty of the 2DLT inhibitor is not its dual activity *per se*, but its potential to inactivate the virus in the absence of cells. For viral entry into cells it is essential that the mousetrap shuts when a mouse is eating from the cheese, in other words when the virus is attaching to a target cell. This timing is coordinated by the activation of Env's spring-loaded fusion machinery only when the virus is attaching to an infectable cell via CD4. It has been previously recognized that CD4 mimetics induce a short-lived activated Env state that deteriorates into an inactive Env form [2]; however, CD4 mimetics are usually not very potent in doing so. The CD4 component of 2DLT also induces the short-lived activated Env form, but then the fusion inhibitor component, T1144, delivers a second blow by binding and blocking the activated fusion machinery in gp41. Thus, 2DLT induces a premature and irreversible collapse of the viral mousetrap, thereby preventing viral entry into target cells. As a consequence, in regular infection inhibition experiments in the presence of target cells, 2DLT is as potent as its most potent constituent, T1144. However, in sharp contrast, 2DLT is able to disable the virus in the absence of target cells, while T1144 is not. What Lu *et al*. [6] did not study is whether the two separate components of 2DLT, T1144 and soluble CD4, can disable the virus when simply mixed. This would reveal whether the combined action of the two components of 2DLT require a physical linkage.

Despite the promise of its novel mechanism of action, some formidable challenges lie ahead before 2DLT or 2DLT-derivates will be suitable for wide clinical use. Poor bioavailability and expensive manufacturing put 2DLT at a disadvantage compared to currently available small molecule inhibitors. However, its mechanism of viral deactivation away from cells puts it at a unique advantage, and as such it warrants further research. 2DLT may be considered for use as a microbicide - for example, in vaginal gels that are aimed at preventing HIV-1 transmission at the vaginal mucosal surface. Another interesting application of 2DLT could be in viral immune prophylaxis (VIP). VIP is a gene therapy vaccination approach in which the constitutive expression by host cells of a neutralizing antibody or an inhibitory protein provides vaccine-like protection against viral infection [9]. In summary, the bi-specific and dual active 2DLT inhibitor described by Lu *et al*., with its one-two punch that inactivates free virus, represents a novel drug approach that warrants further evaluation.
